# Draft Genome Sequences of Probiotic Candidate Schleiferilactobacillus harbinensis Isolated from Fermented Milk “*Laban*”

**DOI:** 10.1128/mra.01043-22

**Published:** 2022-12-06

**Authors:** Abdullah Mashraqi, Behailu B. Eshetea, Broderick Eribo

**Affiliations:** a Biology Department, College of Science, Jazan University, Jazan, Saudi Arabia; b Glidewell Laboratories, Irvine, California, USA; c Department of Biology, Howard University, Washington, DC, USA; University of Maryland School of Medicine

## Abstract

Lactic acid bacteria are a major component of dairy products, especially species belonging to the genus *Lactobacillus*. This study reports the whole-genome sequence of Schleiferilactobacillus harbinensis isolated from *laban*, indigenous fermented milk of Saudi Arabia. The genome sequence is 3,023,618 bp long, has 179 contigs, and has a G+C content of 53.3%.

## ANNOUNCEMENT

Lactic acid bacteria (LAB) play a significant role in the food and health industry for their probiotic potential and antimicrobial properties. In this study, Schleiferilactobacillus harbinensis, formerly Lactobacillus harbinensis, was isolated from fermented milk, *laban*. This species was first isolated from traditional fermented vegetables in China (1) and later from the dairy system, animals, and silage (2, 3). In this study, Schleiferilactobacillus harbinensis was sequenced to uncover the genomic makeup of the isolated probiotic candidate LAB from fermented milk, *laban*.

*Laban* samples, obtained from retail stores in the Washington, DC (USA), area, were serially diluted; plated on De Man, Rogosa, and Sharpe (MRS) agar; and incubated anaerobically for 72 h at 37°C, followed by a random selection of isolates. Schleiferilactobacillus harbinensis was identified by 16S rRNA sequencing (accession number MW662619). A fresh culture was incubated anaerobically on MRS broth at 37°C for 72 h. The pellet from this culture was sent to Genewiz (NJ) for library preparation and whole-genome sequencing (WGS). After the extraction and quantification of the DNA using a Qubit fluorometric spectrophotometer, the DNA library was prepared using the Nextera DNA sample preparation kit, and paired-end sequences (2 × 150 bp) were generated from the Illumina MiSeq platform. The sequences were obtained as a FASTQ file, and the sequence quality was checked using FastQC software (version 0.11.9) (4). The filtered sequences were assembled using Unicycler (version 1) in the Pathosystems Resource Integration Center (5, 6). The identification of the isolate was confirmed using WGS. The annotation was carried out using NCBI Prokaryotic Genome Annotation pipelines (PGAP) (version 5.1) (7, 8). The existence of bacteriocin genes was examined using PGAP, BAGEL4 (9), and antiSMASH (version 5) (10) platforms. PlasmidFinder (version 2.0.1), VirulenceFinder (version 2.0.3), and ResFinder, in the Center for Genomic Epidemiology, were used to assess the presence of plasmids, virulence factors, and antibiotic resistance genes, with a >70% identity (11–13). Default parameters were used for all software except where otherwise noted.

Genome sequences of the isolate were obtained from 1,464,744 reads with a coverage of 178×. The assembly resulted in a genome size of 3,023,618 bp distributed in 179 contigs, with a G+C value of 53.3%, and the contig *N*_50_ value was 43,550 bp. The size of the shortest contig was 302 bp, while the longest was 222,116 bp. The annotation revealed 2,902 protein-coding sequences, including 57 tRNAs, 3 rRNAs, 4 noncoding RNAs, and 58 pseudogenes. Several probiotic-related genes and protein-encoding genes putatively involved in adaptation to different stress conditions were predicted. None of the bacteriocin genes were detected except the bacteriocin immunity protein (GenBank MBO3093097.1), similar to the immunity protein of pediocin-like bacteriocins. Moreover, no plasmid, antibiotic resistance genes, or virulence factors were detected in the genome, confirming the safety of the isolate.

### Data availability.

This whole-genome shotgun project (BioProject PRJNA714415) of Schleiferilactobacillus harbinensis has been deposited to GenBank under the accession number JAGFBC000000000. The version described in this paper is the first version, JAGFBC010000000. The Sequence Read Archive (SRA) submission is available under accession number SRR21672388.
